# Quality of Life After Vestibular Schwannoma Surgery: A Question of Perspective

**DOI:** 10.3389/fonc.2021.770789

**Published:** 2022-02-11

**Authors:** Miriam Bender, Marcos Tatagiba, Alireza Gharabaghi

**Affiliations:** Department of Neurosurgery and Neurotechnology, University Hospital and Faculty of Medicine, University of Tübingen, Tübingen, Germany

**Keywords:** vestibular schwannoma (acoustic neuroma), microsurgery methods, Quality of Life, depression, vertigo, Medical Outcomes Study 36-Item Short Form (SF-36), Beck Depression Inventory (BDI), Dizziness Health Inventory (DHI)

## Abstract

**Objective:**

Health-related quality of life (HRQoL) and self-reported outcome measures have a relevant impact on the medical decision-making process. They capture either the current status and allow for multiple prospective evaluations in the course of a treatment or rely on the retrospective comparison of health of patients before and after an intervention to assess its benefit. Importantly, these patient-assessed measures may be influenced by psychological factors. We compared HRQoL and perceived benefit in the course of surgical vestibular schwannoma (VS) treatment, as assessed by the patients from a prospective and retrospective point-of-view, and evaluated the influence of co-morbid depression.

**Methods:**

Within a prospective observational single-center study, forty-three patients with VS were investigated before and after retrosigmoid tumor resection. SF-36, Beck Depression Inventory and patient-assessed clinical symptoms were acquired before surgery and at follow-up. At follow-up, the Glasgow Benefit Inventory (GBI) was acquired as well.

**Results:**

SF-36 scores were significantly lower than the age and sex matched normative data in six and three out of eight categories before and after surgery, respectively. Three categories improved significantly after vs. before surgery; one of them (global health) reached a minimal clinical important difference. In contrast, patients reported predominantly a deterioration, when asked for a retrospective evaluation of the benefit (i.e., GBI). Depression correlated with both SF-36 and GBI, determined dissatisfaction, improved significantly after surgery and was the measure that had the largest impact on HRQoL.

**Conclusion:**

Prospective and retrospective HRQoL measures may lead to different findings and can be confounded by psychological factors.

## Introduction

Since the goals of vestibular schwannoma (VS) treatment have evolved from preservation of life to tumor control, preservation of facial nerve function and hearing status; health-related quality of life (HRQoL) is increasingly being used as an instrument for outcome-monitoring. HRQoL may be a helpful tool in overcoming the frequently observed inconsistency between objective evaluations of outcomes by medical staff and subjective perception of patients (1). At the same time, HRQoL measures are also used to compare different treatment modalities of VS management and may, thereby, influence the medical decision-making process with regard to conservative management (CM) with interval imaging and regular follow-up, stereotactic radiation therapy (SRT) or microsurgical removal (MS). It is, therefore, particularly important to identify potential influencing factors and possible methodological caveats.

Recently, a systematic review evaluated the impact of different management strategies on HRQoL in VS patients, and summarized the findings of ten prospective and twenty-nine retrospective studies (1). The vast majority of these studies applied either the Short Form 36-items Healthy Survey (SF-36) (2), which captures the current health status, or the Glasgow Benefit Inventory (GBI) (3) that relies on the retrospective comparison of health before and after an intervention to assess its benefit. The retrospective studies that evaluated HRQoL after MS would suggest that MS had a significant negative impact on HRQoL, i.e., that HRQoL deteriorated after surgery when using the SF-36 once after the intervention and comparing the findings with the general population, or when using the GBI after the intervention and asking the patients for a retrospective evaluation of the benefit in comparison with the pretreatment status.

However, prospective studies that applied the SF-36 before and after MS showed a trend towards improved HRQoL (when comparing the post- to the preoperative SF-36 findings). How can this discrepancy between prospective and retrospective studies be explained? In the present study, we intended to address this question by comparing HRQoL and perceived benefit of MS, assessed from both a prospective and retrospective point-of-view, in the same patient population.

Furthermore, the mentioned patient-assessed measures may be influenced by psychological factors. Specifically, depression has been shown to have a negative impact on HRQoL (4), and the SF-36 does indeed include several related questions such as “Have you felt so down in the dumps that nothing could cheer you up? Have you felt downhearted and blue? Did you feel worn out? Have you been a happy person?” among others. Therefore, VS experts have previously considered it ideal to hand out other questionnaires to patients that focus on depression, such as the Beck Depression Index (BDI); at the same time they had concerns that the additional length of the questionnaire and the time required to fill it out would lower the response rate (5). Nonetheless, it was proposed that HRQoL studies in VS patients should include a measure of depression to determine interrelationships and develop strategies for interventions in patients in need (6). However, there is currently no HRQoL study in VS patients that evaluated prospectively, both before and after surgery, the influence of co-morbid depression. This study intended to close this gap.

## Methods

This single-center prospective study was conducted over a 24-month period after approval by the local institutional review board. We invited consecutive VS patients who were scheduled for microsurgical tumor resection *via* the retrosigmoid approach to participate in this observational study. After written informed consent, patients were asked to fill out different questionnaires before surgery and at the first follow-up several months after discharge (mean 7.2 months, min–max: 2–18 months). This study focused on this medium-term follow-up, instead of long-term follow-ups in the order of years, to allow for recovery from surgery, but minimize at the same time the patients’ memory distortions in the context of their retrospective evaluation of their preoperative status (see the *Discussion* section for further details). Additionally, we recorded clinical characteristics such as age, sex, tumor location and size (Hannover classification) (7). The occurrence of meningitis, cerebrospinal fluid leak, hemorrhage, new cerebrospinal fluid (CSF) circulation disorder, or the need for revision were considered postoperative complications.

Prospectively, the questionnaires assessed quality of life with the Short Form 36-items Healthy Survey (SF-36) (2, 8), depression with the Beck Depression Inventory (BDI) (9) and vertigo with the Dizziness Handicap Inventory (DHI) (10). Retrospectively, the Glasgow Benefit Inventory (GBI) was used at follow-up. It relies on the retrospective comparison of health of the patients before and after an intervention to assess its benefit. Clinical characteristics like hearing function and facial nerve function were assessed with a self-reported five point scale, ranging from 0 (not affected) to 5 (very severely affected), while headache and dizziness were rated on a visual analog scale (range 0–100). Further methodological details with regard to the applied questionnaires can be found in the supplemental digital content.

Statistical analysis was performed with SPSS software (SPSS for Windows, version 15.0: SPSS, Inc., Chicago, IL). A level of significance at α = 0.05 was applied. For correlations, Spearman-Rho (r_s_) was calculated for a robust estimation even in case of non-normal distribution. For comparing group differences chi² test was used for categorical variables and a Mann–Whitney–U test for at least ordinal scaled variables. To compare the change in depressive patients before and after surgery McNemar test was used.

## Results

Forty-three patients were included in the data evaluation after excluding nine NF-2 patients and nine patients with incomplete questionnaires and/or loss to follow-up. Demographics, clinical information and patient-assessed functional parameters are shown in **Table 1** and **Supplemental Online Table 1**, respectively. Postoperative complications occurred in two patients (hemorrhage requiring surgical revision and a CSF leak that could be treated by spinal CSF drainage), with one case in the depressive subgroup. Neither of the two cases was an outlier in any of the collected scores. Due to the low number of cases with postoperative complications, a subgroup analysis could not be performed. Our patient population had a significantly larger point prevalence of depression than the German general population (8%) (11), both before (23%, χ² (1) = 13.32, p <.001) and after (17%, χ² (1) = 4.25, p = .03) surgery. The rate of depressive patients dropped significantly after surgery (exact McNemar, p <.001). Depressive patients had a significantly higher DHI score (**Table 1**).

**Table 1:** Demographics and clinical data.

	all	BDI ≥10	BDI <10	
Age (years), mean (min–max)	48.9 (22–76)	52.9 (36–72)	47.7 (22–76)	n.s. (Mann–Whitney–U)
Sex	24 females, 19 males	6 females, 4 males	18 females, 15 males	n.s. (Chi²)
Affected side	28 left, 15 right	6 left, 4 right	22 left, 11 right	n.s. (Chi²)
Tumor size* (Hannover classification)				n.s. (Mann–Whitney–U)
T1	6	0	6	
T2	10	2	8	
T3a	4	2	2	
T3b	9	0	9	
T4a	11	6	5	
T4b	2	0	2	
Depression (BDI ≥10)**	10 from 43 (23%) follow-up: [7 from 42 (17%)]	–	–	
Mean DHI Score (SD)	24.2 ( ± 25.1)	42.6 ( ± 32.9)	18.9 ( ± 19.6)	0.039 (Mann–Whitney–U)

*Not recorded for one patient; **One patient did not return the BDI at follow-up; n.s., not significant.

**Figure 1:**
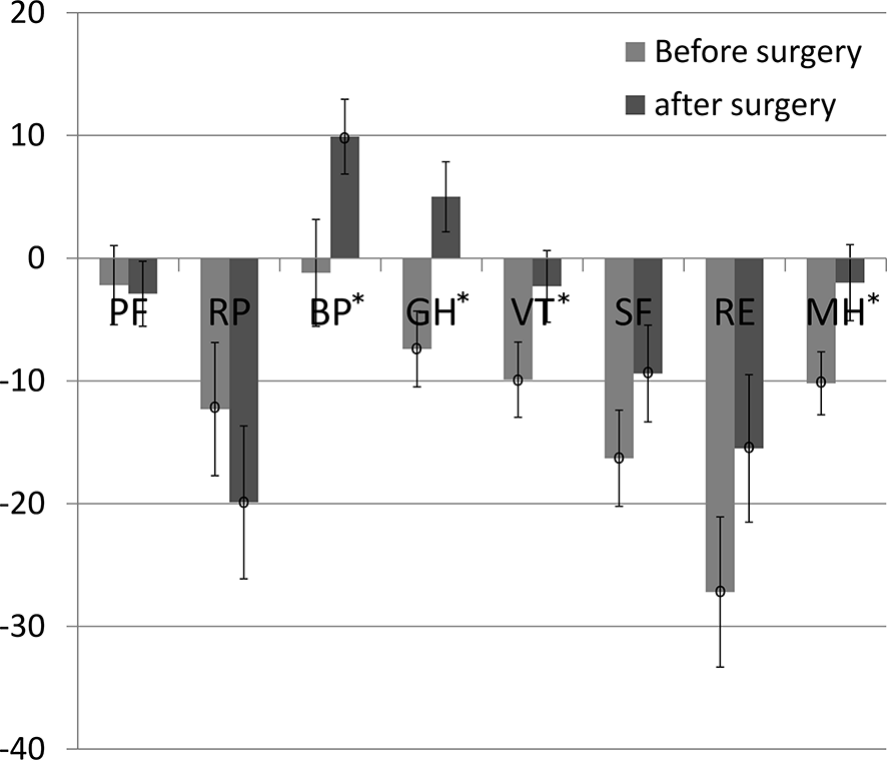
SF-36 pre- and post-data in comparison to normative data. Significant changes from before to after surgery have been marked with “*”; significant differences compared to the German normative data have been marked with “°” at the center of the error bar, which reflects the standard error.

**Figure 2:**
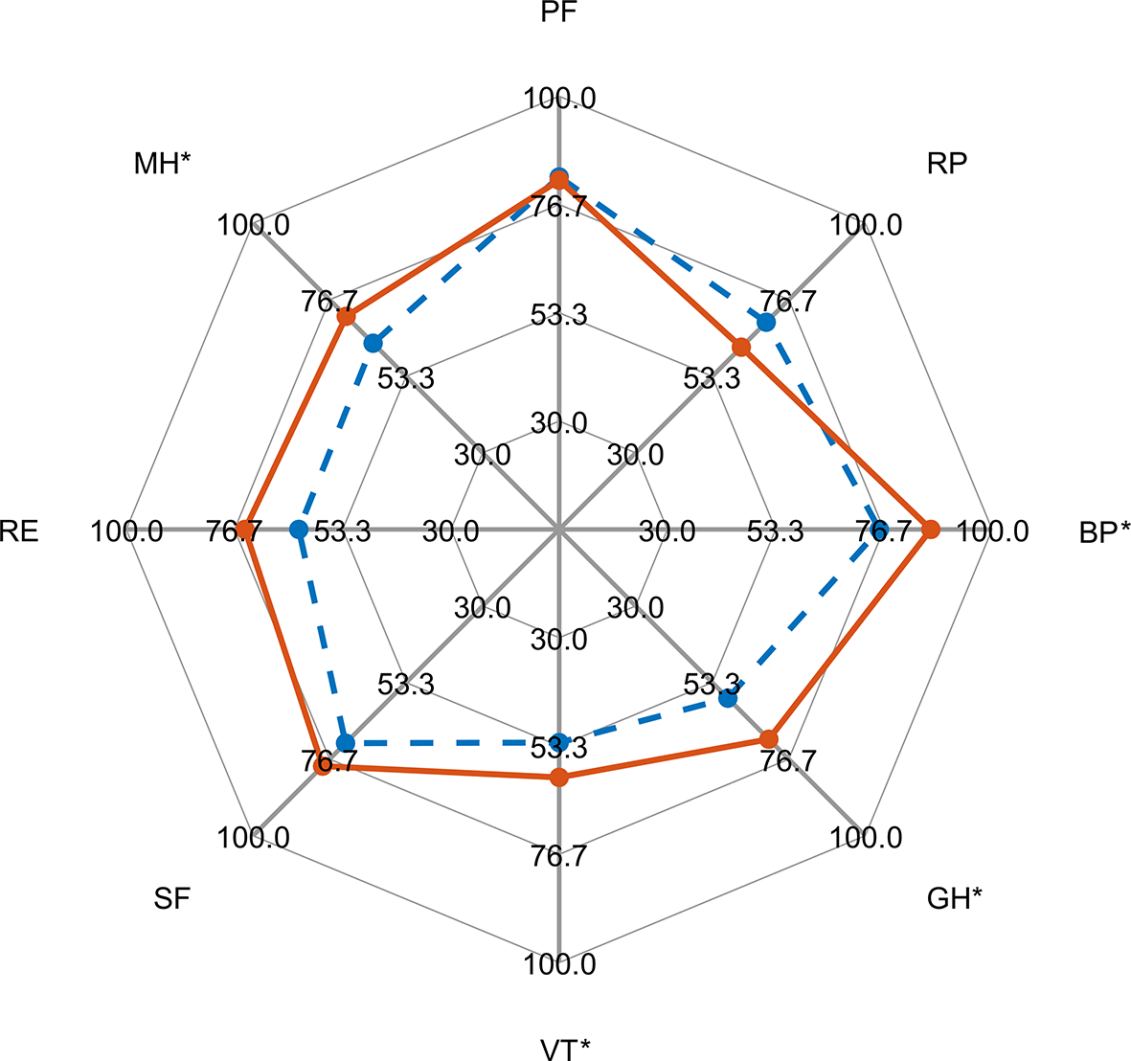
Radar graph of SF-36 raw values before surgery (blue dashed line) and at follow-up (red solid line). Domains with a significant difference (paired t-test) are marked with an asterisk.

The HRQoL, i.e., SF-36 scores, were significantly lower than the age and sex matched normative data in six (RP, GH, VT, SF, RE, and MH) and three (RP, SF, RE) out of eight categories before and after surgery, respectively (see **Supplemental Online** **Table 2**). Three categories (BP, GH, MH) improved significantly after surgery in comparison to the preoperative status (see **Figure 1**). Only one of them (GH) reached a minimal clinical important difference (MCID) and a large enough responsiveness for the estimation of a valid MCID (**Table 2**, **Figure 2**).

**Table 2:** Post- vs. pre-comparison of SF-36.

SF-36 category	Patients (N = 43) before surgery	Patients (N = 43) at follow-up	Mean change	Mean change 95% -CI	Effect size	Paired t-test before surgery vs. follow-up	MCID
Physical functioning (PF)	82.6 ± 23.9	81.9 ± 20.7	−0.7	-7.1–5.7	0.03	t (42) = 0.22 p = 0.827	6.0
Role functioning—physical (RP)	69.8 ± 36.4	62.2 ± 42.7	-7.6	−22.5–7.4	0.21	t (42) = 1.02 p = 0.314	16.6
Bodily pain (BP)	75.7 ± 29.7	86.8 ± 20.4	11.1	3.0–19.1	0.37	**t (42**) **=** −**2.76** **p = 0.008**	12.7
General health (GH)	58.1 ± 19.6	70.6 ± 18.2	**12.4**	5.8–19.1	0.63	**t (42**) **=** −**3.77** **p = 0.001**	10.2
Vitality (VT)	52.6 ± 20.8	60.1 ± 19.6	7.6	0.7–14.4	0.36	**t (42**) **=** −**2.23** **p = 0.031**	7.8
Social functioning (SF)	71.8 ± 26.0	78.8 ± 26.2	7.0	−2.0–16.0	0.27	t (42) = −1.56 p = 0.126	12.1
Role functioning—emotional (RE)	62.8 ± 40.6	74.4 ± 40.4	11.6	−1.4–24.7	0.29	t (42) = −1.80 p = 0.079	16.2
Mental health (MH)	63.4 ± 16.9	71.6 ± 19.8	**8.2**	2.4–14.0	0.48	**t (42**) **=** −**2.85** **p = 0.007**	6.2
DHI	24.2 ± 25.1	24.2 ± 24.0	0.0	−6.5–6.5	0.00	t (42) = 0.00, p = 1	–
BDI	6.5 ± 5.6	4.1 ± 4.4	2.4	1.0–3.8	0.43	**t (42**) **= 3.41, p = 0.001**	–

Significant differences have been marked bold in the ‘t-test’ column, while in the ‘Mean change’ column only differences above the minimal clinical important difference (MCID) have been marked bold.

**Figure 3:**
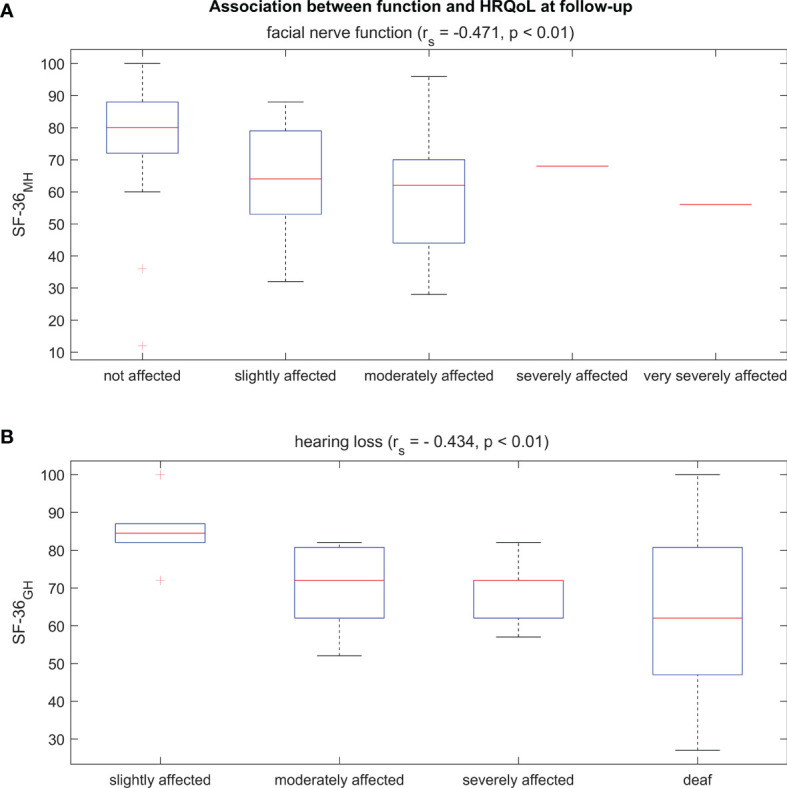
Boxplots of the SF-36 scores of two selective domains that reached a significant correlation at follow-up with facial nerve function **(A)** and hearing loss **(B)**.

The HRQoL was significantly determined by depression; i.e., depressive patients had significantly worse total SF-36 scores at baseline and follow-up than non-depressive patients (p <0.001, **Table 3**). Accordingly, correlation analysis (see **Supplemental Online Table 3**) revealed strong associations between almost all SF-36 scores and BDI scores (7/8 subscores, max. r_s_ = −0.878), DHI scores (7/8 subscores, max. r_s_ = −0.830), and self-reported dizziness (6/8 subscores, max. r_s_ = −0.563), and only weak correlations in some SF-36 subscore with self-reported facial nerve paresis (3/8, max. r_s_ = −0.471, see **Figure 3A**) and hearing loss on the affected side (2/8, max. r_s_ = −0.434, see **Figure 3B**) at follow-up. Deterioration in the two clinical functional variables (facial nerve function and hearing loss on the affected side) did not correlate significantly with the change in SF-36 subscores (see **Supplemental Online Table 6**).

**Table 3:** SF-36 subgroup analysis, median (25^th^ 75^th^ percentile) and results of Mann-Whitney-U test are given for each subscore of the SF-36.

	PF		RP		BP		GH		VT		SF		RE		MH	
**Baseline**																
Female, N = 24	-1.9 (-14.2 9.9)	Z=-1.163, p=.250	-17.7 (-45.5 11.0)	Z =-1.494, p=.138	-3.4 (-27.1 18.3)	Z=-0.944, p=.352	-2.8 (-24.9 8.9)	Z=-0.232, p=.823	-17.7 (-22.9 -3.4)	Z=-0.905, p=.373	-18.6 (-36.8 3.1)	Z=-0.441, p=.667	-54.7 (-57.2 9.4)	Z=-0.380, p=.712	-9.3 (-26.1 -1.5)	Z=-0.367, p=.721
Male, N = 19	4.5 (-0.5 9.5)		12.0 (-20.8 16.7)		19.2 (-6.8 19.2)		-14.5 (-25.9 4.1)		-7.3 (-20.8 14.2)		-5.6 (-38.4 8.9)		7.6 (-59.1 10.0)		-12.0 (-20.5 2.9)	
**Follow up**																
Female, N = 24	-0.4 (-18.3 5.5)	Z=-1.236, p=.221	-14.0 (-72.7 11.9)	Z=-1.384, p=.170	15.7 (-7.8 29.6)	Z=-0.049, p=.966	2.9 (-9.1 23.4)	Z=-0.269,p=.795	-2.3 (-15.1 2.5)	Z=-0.342, p=.739	-6.3 (-31.4 12.8)	Z=-0.490, p=.632	9.4 (-74.9 12.0)	Z=-0.872, p=.390	0.5 (-10.7 13.4)	Z=-0.245, p=.813
Male, N = 19	3.6 (-5.5 9.5)		8.6 (-38.0 12.0)		19.2 (-5.6 24.3)		13.1 (-16.9 14.9)		1.5 (-15.8 14.2)		-1.3 (-25.9 8.9)		7.6 (-25.7 10.0)		-0.0 (-8.5 13.3)	
**Baseline**																
T1/T2, N = 16	6.3 (-1.4 9.5)	Z=-1.037, p=.306	11.0 -15.0 15.5)	Z=-1.335, p=.186	17.4 (-10.6 19.2)	Z=-0.961, p=.344	-4.8 (-27.4 8.9)	Z=-0.246, p=.813	-14.9 -21.1 11.0)	Z=-0.181, p=.863	-2.2 (-25.6 8.9)	Z=-1.426, p=.157	6.4 (-81.3 9.6)	Z=-0.039, p=.974	-6.5 -20.4 -0.3)	Z=-0.505, p=.622
T3/T4, N = 26	0.9 (-17.9 9.7)		-17.9 (-41.2 12.3)		3.4 (-25.5 21.4)		-10.5 (-23.6 5.2)		-16.2 (-23.6 5.2)		-24.9 (-37.8 4.5)		-40.2 (-57.2 9.6)		-11.1 (-24.8 -1.1)	
**Follow up**																
T1/T2, N = 16	3.6 (-15.1 8.6)	Z=-0.026, p=.985	-11.2 (-76.4 11.1)	Z=-1.245, p=.218	13.4 (-7.5 19.2)	Z=-1.181, p=.243	10.5 (-11.7 14.7)	Z=-0.130, p=.903	0.2 (-16.4 9.2)	Z=-0.427, p=.677	7.9 (-24.8 11.0)	Z=-0.506, p=.621	7.6 (-26.5 9.6)	Z=-0.950, p=.350	2.0 (-8.4 13.0)	Z=-0.324, p=.754
T3/T4, N = 26	0.9 (-17.5 10.9)		-0.1 (-64.5 15.4)		15.9 (-2.2 29.6)		4.0 (-10.1 23.3)		-2.7 (-15.8 8.2)		-3.6 (-34.4 12.6)		9.4 (-55.8 11.0)		-1.0 (-11.7 10.8)	
**Baseline**																
BDI_baseline_ < 10, N = 33	3.6 (-2.8 9.5)	Z=-1.554, p=.123	8.6 (-32.4 14.3)	Z=-1.555, p=.123	14.2 (-14.9 21.7)	**Z=-2.479, p=.012**	**-1.1 (-21.1 9.5)**	**Z=-2.401, p=.015**	-8.5 (-21.7 6.7)	Z=-1.495, p=.139	**-5.6 (-26.2 8.9)**	**Z=-2.793, p=.004**	6.4 (-55.3 9.7)	Z=-1.871, p=.062	**-3.8 (-15.1 2.7)**	**Z=-3.364, p<.001**
BDI_baseline_ ≥ 10, N = 10	-17.3 (-41.7 10.9)		-30-4 (-66.1 11.5)		**-42.3 (-55.0 16.6)**		**-22.0 (-34.6 -15.8)**		-20-6 (-23.9 -12.5)		**-37.9 (-57.0 -12.9)**		-69.5 (-89.4 8.2)		**-26.7 (-35.2 -16.5)**	
**Follow up**																
BDI_baseline_ < 10, N = 33	2.7 (-5.5 7.8)	Z=-0.992, p=.330	-2.8 (-47.6 12.0)	Z=-0.662, p=.518	15.7 (-6.2 27.9(	Z=-0.058, p=.960	13.1 (-4.2 21.1)	Z=-1.927, p=.054	**1.5 (-10.3 12.1)**	**Z=-2.185, p=.028**	7.9 (-22.4 11.9)	Z=-1.829, p=.068	**7.6 (-8.1 10.5)**	**Z=-2.007, p=.044**	**4.0 (-5.9 14.4)**	**Z=-2.171, p=.029**
BDI_baseline_ ≥ 10, N = 10	-14.7 (-23.6 10.7)		-42.1 (-74.0 13.2)		15.7 (-9.6 25.6)		-4.6 -26.1 14.1)		**-15.4 (-34.1 -1.6)**		-35.7 (-61.4 11.9)		**-84.8 (-90.0 10.4)**		**-13.5 (-42.4 4.0)**	
*BDI_follow-up_ < 10, N = 35	0.2 (-17.3 9.5)	Z=-0.304, p=.773	**4.2 (-57.2 12.3)**	**Z=-2.3, p=.022**	15.7 (-6.8 27.9)	Z=-0.423, p=.685	**13.5 (-4.5 23.2)**	**Z=-2.330, p=.009**	**1.5 (-8.1 9.9)**	**Z=-3.646, p<.001**	**7.9 (-12.5 12.8)**	**Z=-3.770, p<.001**	**7.6 (-22.6 10.7)**	**Z=-1.982, p=.047**	**4.9 -5.6 14.9)**	**Z=-3.528, p<.001**
*BDI_follow-up_ ≥ 10, N = 7	1.7 (-22.3 5.9)		**-72.3 (-89.0 -8.3)**		15.7 (-18.8 24.3)		**-10.1 (-24.0 9.1)**		**-28.1 (-37.3 -15.8)**		**-37.2 (-63.8 -35.3)**		**-89.3 (-90.0 10.3)**		**-39.8 (-50.2 -6.2)**	

*One patient did not return the BDI at follow-up, signficiant results have been marked bold.

Despite the HRQoL improvement that was documented with the prospective SF-36 evaluation, patients reported predominantly deterioration, when asked for a retrospective evaluation of the benefit after surgery (i.e., GBI, **Table 4**). This negative assessment was significantly accentuated in depressive patients (BDI ≥10) for the general subscore. The total GBI score was significantly correlated (see **Supplemental Online** **Table 4**) with self-reported facial nerve paresis (r**_s_** = −0.433), hearing loss (r_s_ = −0.418) and BDI-score (r_s_ = −0.494), but showed only a weak correlation with DHI-score (r_s_ = 0.308) and no significant correlation with self-reported dizziness. The GBI total score showed a better correlation with the postoperative function measurements and SF-36 score than with the change in these measurements at follow-up compared to the preoperative status.

**Table 4:** GBI with subgroup analysis, BDI ≥ 10 indicates depression.

	All	BDI ≥ 10	BDI < 10	Mann-Whitney- U
**GBI total**				
Worse	60.5%	80%	54.5%	
Unchanged	9.3%	0%	12.1%	
Improved	30.2%	20%	43.3%	
Median (25^th^ 75^th^ percentile)	-5.6 (-13.2 2.8)	-11.1 (-25.7 -2.8)	-2.8 (-11.8 3.5)	Z=-1.939, p=.052
**General**				
Worse	67.4%	90%	60.6%	
Unchanged	11.6%	0%	15.2%	
Improved	21.0%	10%	24.2%	
Median (25^th^ 75^th^ percentile)	-12.5 (-29.2 0)	**-20.8 (-39.6 -10.4)**	**-8.3 (-26.0 1.0)**	**Z=-2.098, p=.035**
**Social**				
Worse	0%	0%	0%	
Unchanged	34.9%	20%	39.4%	
Improved	65.1%	80%	60.6%	
Median (25^th^ 75^th^ percentile)	16.7 (0 33.3)	33.3 (12.5 37.5)	16.7 (0 33.3)	Z=-0.722, p=.475
**Physical**				
Worse	39.5%	50%	36.4%	
Unchanged	46.5%	20%	54.5%	
Improved	14.0%	30%	9.1%	
Median (25^th^ 75^th^ percentile)	0.0 (-16.7 0)	-8.3 (-16.7 20.8)	0 (-16.7 0)	Z=-1.407, p=.161

Signficiant results have been marked bold.

Depression was the only measure that showed a constantly high correlation with both the SF-36 and the GBI; moreover, the mean BDI score showed a significant improvement after vs. before surgery (p <0.001, **Table 2**).

## Discussion

### Health-Related Quality of Life in Vestibular Schwannoma Patients

This study revealed a significantly reduced HRQoL of VS patients in comparison to age and sex matched normative data already before surgery. Notably, this was true for a mixed group of patients with VS of all sizes, and not for a selected sample of large tumors only (12). This preoperatively reduced HRQoL is in line with previous prospective studies that also described significantly lower HRQoL before surgery compared with the general population (12–14). Together, these observations indicate that snapshots taken after an intervention, e.g., applying the SF-36 after surgery only without preoperatively evaluating a baseline condition, may lead to erroneous conclusions in the form of attributing reduced HRQoL in comparison to the general population to the intervention. This may explain the discrepancy between prospective and retrospective studies in the past (1). Therefore, many studies that investigated the HRQoL retrospectively by applying the SF-36 only after an intervention should be interpreted cautiously. Even when comparing different treatment modalities with this method, the observations may be misleading due to potentially different baseline values before the respective interventions. This may be particularly true for comparisons between an active intervention (MS or SRT) and an observational approach (with regular follow-ups and interval imaging), since these patient groups may already differ relevantly in their baseline HRQoL before they make a decision for one treatment approach or the other. Specifically, patients who feel less compromised in their health-related quality of life, and therefore have higher HRQoL levels at baseline, may be more hesitant to opt for a potentially risky intervention. These patients will then likely have higher HRQoL levels later on as well, when compared to patients who underwent active interventions. Prospective evaluations with baseline HRQoL assessments that are acquired before the respective therapeutic interventions are therefore mandatory before comparing different treatment modalities and drawing conclusions.

### Improvement of Health-Related Quality of Life After Surgery

Of the six SF-36 categories in our study that were significantly lower than the age and sex matched normative data before surgery, three improved significantly in comparison to the preoperative status and reached the levels of the disease-free population. The category *general health* achieved a robust minimal clinical important difference, while the category *bodily pain* reached even a level that was significantly higher than the normative data (**Figure 1**). The latter finding may be interpreted along the lines of previous explanations by the recovery from the trauma of surgery, and with increased social support and tolerance of pain following the experience of an operation (1). Notably, we observed this HRQoL improvement at a relatively early follow-up visit (i.e., on average 7 months after surgery), while previous work reported an initial postoperative decline, before a HRQoL improvement ensued during the following years (14–16). The reported HRQoL categories that improved in these studies and ours are quite heterogeneous suggesting a large variability of the perceptions and recovery patterns of the patients.

The SF-36 changes in our study, mainly correlated with DHI and self-reported dizziness, i.e., the less vestibular symptoms the better was the HRQoL, which is in line with previous VS studies [e.g., (17–19)]. There was only a small effect of hearing loss and facial paralysis on some categories of the SF-36, which is also in line with previous VS studies [e.g., (19, 20)]. Notably, more disease specific questionnaires such as the validated Penn Acoustic Neuroma Quality of Life Scale [PANQOL (21)], show a strong difference between healthy controls and VS patients for the facial function and hearing subdomains (18). This emphasizes the need to use disease specific questionnaires. However, the collection of our patient cohort started before this questionnaire was available for our German-speaking patients (22).

### Retrospective Evaluation of the Benefit From Surgery

In contrast to the findings of the prospective HRQoL assessments in our study, patients reported predominantly a deterioration, when asked for a retrospective evaluation of the benefit from surgery *via* the GBI (**Table 4**) in accordance with the results of a recent study that examined QoL on average 7.7 years after VS resection (23). This may have different, mutually non-exclusive explanations. The GBI correlated with the self-reported facial nerve paresis and hearing loss and may therefore have captured different aspects of HRQoL that were missed by the SF-36. These generic questionnaires have also previously been shown to examine subtly different areas of function (1). Furthermore, these GBI findings relatively early after surgery may have reflected the initial postoperative decline reported in earlier work (and mentioned in the previous section), before recovering again in the course of the following years (14–16).

A complementary potentially more informative explanation may be provided by the observations of another study. In a large sample of VS patients (768 operated vs. 247 observed) that was retrospectively evaluated, the same proportion of patients (37% vs. 38%) considered hearing loss as the worst aspect of their treatment choice regardless of being operated or observed (24). Moreover, similar proportions in both groups were not satisfied with the information provided by the medical staff (38% vs. 24%) or even regretted the choice they made (8% vs. 5%). We suggest that these observations open up an interesting perspective of potential cognitive mechanisms: When patients look back on their decision regarding a treatment option, they link general disease-related symptoms that may occur independent of the treatment option with their specific decision. This may lead to dissatisfaction and even regret with the decision made.

Moreover, many patients feel—in retrospect—insufficiently informed by the medical staff, which may reflect ineffective informed consent, unmet communication and information needs and/or patients’ memory distortions in the process of informed consent. More specifically, patients are likely to forget exact information that they have been told, even if understood at the time (25). Even if patients could remember exact information, they are likely to base decisions on independent gist representations. Clear communication of risks is a necessary precondition for accurate patient perceptions, but seems often not sufficient (25).

Along these lines, perceived benefit and quality of life following vestibular schwannoma surgery (and any treatment decision in general) may be influenced by memory distortions of patients as well, when evaluated retrospectively. Measures such as the Glasgow Benefit Inventory (GBI), for example, imply that the baseline value is zero and rely on the pre- and postoperative retrospective comparison of health of the patient to assess the benefit of the intervention. These retrospective evaluations may result from forgetting the information provided before the intervention and/or disregarding the limited health status and quality of life before the intervention. Furthermore, cognitive functions and memory may be impaired due the acute situation and/or correspond to co-morbid psychological factors such as depressive symptoms (26).

### The Impact of Depression in Health-Related Quality of Life in Vestibular Schwannoma

Depression correlated inversely with both SF-36 and GBI, determined dissatisfaction, improved significantly after surgery and was the measure that had the largest impact on HRQoL. Importantly, the decreased HRQoL was paralleled by an unexpectedly high proportion of depressive patients in our study population already before surgery (23%). This finding may reassure pervious notions that demanded support for these patients from a psychologist or psychiatrist (27). The level of distress is not necessarily associated with the extend of functional deficits (28), since depression can make coping with a symptom much more difficult and may explain some of the discrepancy between symptoms of impairment and the perceived handicap (6). Because new symptoms resulting from surgery will always be a source of disappointment for these patients (29), we might consider providing professional help even before surgery to improve the conditions for optimal postoperative recovery and functional restoration.

## Conclusions

For the evaluation of HRQoL generic and disease-specific assessment tools should be applied prospectively before and after an intervention, while considering a potential confounding by psychological factors. Interdisciplinary treatment concepts should address depressive symptoms in VS patients before and after interventions.

## Data Availability Statement

The raw data supporting the conclusions of this article will be made available by the authors, without undue reservation.

## Ethics Statement

The studies involving human participants were reviewed and approved by the Ethik-Kommission an der Medizinischen Fakultät der Eberhard-Karls-Universität und am Universitätsklinikum Tübingen (166/2007BO2). The patients/participants provided their written informed consent to participate in this study.

## Author Contributions

All authors contributed to the study conception and design. Material preparation, data collection and analysis were performed by MB. The first draft of the manuscript was written by MB and all authors commented on previous versions of the manuscript. All authors contributed to the article and approved the submitted version.

## Funding

We acknowledge support by the Open Access Publishing Fund of the University of Tübingen.

## Conflict of Interest

The authors declare that the research was conducted in the absence of any commercial or financial relationships that could be construed as a potential conflict of interest.

## Publisher’s Note

All claims expressed in this article are solely those of the authors and do not necessarily represent those of their affiliated organizations, or those of the publisher, the editors and the reviewers. Any product that may be evaluated in this article, or claim that may be made by its manufacturer, is not guaranteed or endorsed by the publisher.
