# Supplementary material for: Quality of Life After Vestibular Schwannoma Surgery: A Question of Perspective
Source: Front Oncol. 2022 Feb 11;11:770789. doi: 10.3389/fonc.2021.770789 (PMC8873590; doi:10.3389/fonc.2021.770789)
Supplement: Supplementary file 1 [file DataSheet_1.pdf]

Supplementary Material

Methodological Details

The SF-36 subscores (uncorrected for age and gender) were used for the following calculations: A paired t-test was used to calculate significant differences between the SF-36 subscores before surgery and at follow-up. Furthermore, we assessed the minimal clinical important difference (MCID) for the SF-36 subscores. The MCID can be defined either anchor-based or distribution-based. Usually, anchor-based methods are preferred since they better incorporate the patients' perspective (Ward MM, Guthrie LC, Alba MI. Clinically important changes in short form 36 health survey scales for use in rheumatoid arthritis clinical trials: the impact of low responsiveness. *Arthritis Care Res (Hoboken).* Dec 2014;66(12):1783-1789). For VS patients, however, anchor-based MCID levels have been defined for the physical and mental health components summary scores only (Carlson ML, Tveiten OV, Yost KJ, Lohse CM, Lund-Johansen M, Link MJ. The Minimal Clinically Important Difference in Vestibular Schwannoma Quality-of-Life Assessment: An Important Step beyond P < .05. *Otolaryngol Head Neck Surg*. Aug 2015;153(2):202-208.). Therefore, a distribution-based method that was previously applied for VS patients (Turel MK, Thakar S, Rajshekhar V. Quality of life following surgery for large and giant vestibular schwannomas: a prospective study. *J Neurosurg.* Feb 2015;122(2):303-311) was used to define MCID in this study as well according to the following calculation: SD of the baseline scores multiplied by the square root of (1 – r), where r is the Cronbach's coefficient α of the subscore at baseline. Additionally, the responsiveness of each subscore was calculated by the effect size (mean change score divided by SD of the baseline score). A minimum effect size of 0.5 was considered to reflect acceptable responsiveness, in accordance with the above mentioned references.

There is a known influence of age and sex on the SF-36. Therefore, also the difference to the age and gender matched normative data of the German population was calculated of each patient (Bullinger M, Kirchberger I. *Der SF-36-Fragebogen zum Gesundheitszustand: Handbuch für die deutschsprachige Frageversion*. Göttingen: Hogrefe Verlag für Psychologie; 1998; Ellert U, Bellach B. Der SF-36 im Bundes-Gesundheitssurvey - Beschreibung einer aktuellen Normstichprobe. *Gesundheitswesen.* 1999;61:184-190). A one-

sample t-test was performed with the resulting value detecting significant deviations from 0 (no difference compared to the age and gender matched normative values). These corrected values were used to investigate the effect of preoperative dizziness, depression, sex and tumor size on the SF-36 with a Mann-Whitney-U test

Patients with a BDI score of ≥10 are considered as depressive (Kendall PC, Hollon SD, Beck AT, Hammen CL, Ingram RE. Issues and recommendations regarding use of the Beck Depression Inventory. *Cognitive Therapy and Research.* 1987;11(3):289-299). For consistency, these patient-assessed read-outs were compared to functional outcome measures that were also assessed by the patients themselves. For this purpose, patients were asked to rate their hearing loss (none, mild, medium, severe, deaf), facial nerve paresis (none, mild, medium, severe, very severe), headache (none, mild, medium, severe, very severe), sleeping problems (none, mild, medium, severe, very severe) and dizziness (10-point visual analogue scale) at both time points as well. Patient assessed functional measures were correlated with SF-36 subscores.

Retrospectively, the Glasgow Benefit Inventory (GBI) was used at follow-up. It relies on the patients' retrospective comparison of health before and after an intervention to assess its benefit. This questionnaire was developed to measure the effect of otorhinolaryngological interventions (Robinson K, Gatehouse S, Browning GG. Measuring patient benefit from otorhinolaryngological surgery and therapy. *Ann Otol Rhinol Laryngol.* Jun 1996;105(6):415-422). It consists of a total score and three subscales (general, social support, physical health) and ranges from +100 (best positive effect), 0 (no effect) to -100 (worst negative effect) after surgery. It was correlated with SF-36 subscores, functional measures at follow-up, and with the change in function outcome measures and in SF-36 subscores between follow-up and preoperative status. As the influence of age and gender on the GBI is unknown, uncorrected SF-36 subscores were used.

Supplemental Online Table 1: Patient-assessed functional parameters

	Before surgery	At follow-up
VAS dizziness (Median, 25th – 75th percentile)	1.5, 0 – 3.5	1.5, 0 – 3.0
Hearing loss		
None	6	0
Mild	10	8
Medium	11	7
Strong	8	6
Deaf	7	19
Facial nerve paresis		
None	39	25
Mild	2	5
Medium	0	8
Strong	2	1
Very strong	0	1
Sleeping problems		
None	28	26
Mild	5	7
Medium	8	5
Strong	2	2

Very strong	0	0
Headache		
None	25	26
Mild	7	6
Medium	5	7
Strong	5	1
Very strong	1	0

Before surgery N = 43, at follow-up N = 40

Supplemental Online Table 2: SF-36 in comparison to normative data

SF-36 category	Mean Difference before surgery (95% -CI)	p (t-test)	Mean Difference follow-up (95% -CI)	p (t-test)
Physical functioning (PF)	-2.2 (-8.7 – 4.3)	0.499	-2.9 (-8.2 – 2.4)	0.280
Role functioning – physical (RP)	**-12.3 (-23.3 – -1.4)**	**0.028**	**-19.9 (-32.5 - -7.3)**	**0.003**
Bodily pain (BP)	-1.2 (-10.0 – 7.6)	0.786	**9.9 (3.7 – 16.0)**	**0.002**
General health (GH)	**-7.4 (-13.6 – -1.1)**	**0.021**	5.0 (-0.7 – 10.8)	0.085
Vitality (VT)	**-9.9 (-16.1 - -3.7)**	**0.002**	-2.3 (-8.2 – 3.6)	0.433
Social functioning (SF)	**-16.3 (-24.4 – 8.5)**	**0.0001**	**-9.4 (-17.4 - -1.5)**	**0.021**
Role functioning – emotional (RE)	**-27.2 (-39.5 – 14.8)**	**<0.0001**	**-15.5 (-27.7 - -3.4)**	**0.013**
Mental health (MH)	**-10.2 (-15.4 - -5.0)**	**0.0003**	-2.0 (-8.3 – 4.2)	0.515

Supplemental Online Table 3 a: Spearman correlation between SF-36 subscores and BDI, DHI and functional measures at before surgery; Signficant < 0.05, Significant <0.01

SF-36	Age	BDI	DHI	Hearing loss	Facial paralysis	VAS Dizziness	Headache
PF	-0.362	-0.436	-0.838	-0.469	-0.281	-0.792	-0.053
RP	-0.169	-0.407	-0.520	-0.231	-0.434	-0.434	-0.008
BP	-0.136	-0.258	-0.433	-0.299	-0.155	-0.421	-0.492
GH	-0.008	-0.609	-0.412	-0.344	0.010	-0.421	-0.120
VT	-0.099	-0.441	-0.369	-0.110	-0.189	-0.399	-0.202
SF	-0.172	-0.624	-0.301	-0.134	-0.076	-0.285	0.119
RE	-0.043	-0.539	-0.172	-0.225	-0.185	-0.100	0.096
MH	0.163	-0.703	-0.160	-0.093	-0.052	-0.179	-0.074

S

Supplemental Online Table 3 b: Spearman correlation between SF-36 subscores and BDI, DHI and functional measures at follow up; Signficant < 0.05, Significant <0.01

SF-36	Age	BDI	DHI	Hearing loss	Facial paralysis	VAS Dizziness	Headache
PF	-0.488	-0.453	-0.830	-0.092	-0.209	-0.563	-0.142
RP	-0.296	-0.472	-0.543	0.085	0.045	-0.445	-0.160
BP	-0.075	-0.151	-0.262	-0.081	-0.076	-0.198	-0.513
GH	-0.047	-0.574	-0.402	-0.434	-0.178	-0.187	-0.082
VT	-0.201	-0.878	-0.479	-0.228	-0.296	-0.349	-0.223
SF	-0.328	-0.761	-0.548	-0.140	-0.380	-0.479	-0.030
RE	-0.173	-0.461	-0.338	0.010	-0.315	-0.495	-0.145
MH	-0.164	-0.717	-0.424	-0.346	-0.471	-0.423	-0.216

Supplemental Online Table 4: Spearman correlation between GBI scores and BDI, DHI, SF-36 subscores (uncorrected) and functional measures at follow up ; Signficant < 0.05, Significant <0.01

	Age	BDI	DHI	Hearing loss	Facial paralysis	VAS Dizziness	Headache	
GBI total	-0.096	-0.494	-0.308	-0.418	-0.433	-0.165	-0.099	
GBI general	-0.160	-0.585	-0.362	-0.379	-0.504	-0.251	-0.110	
GBI social support	0.366	0.394	0.248	-0.143	0.142	0.208	0.055	
GBI bodily health	-0.127	0.035	0.017	-0.123	-0.092	0.111	-0.134	
	PF	RP	BP	GH	VT	SF	RE	MH
GBI total	0.299	0.350	0.086	0.541	0.523	0.393	0.262	0.473
GBI general	0.359	0.350	0.127	0.576	0.587	0.472	0.318	0.507
GBI social support	-0.180	-0.112	-0.186	-0.212	-0.214	-0.265	-0.060	-0.111
GBI bodily health	-0.047	0.204	0.130	0.189	0.110	-0.125	-0.156	-0.065

Supplemental Online Table 5: Spearman correlation between GBI scores and the change in BDI, DHI, SF-36 subscores (uncorrected) and functional measures between baseline (before surgery) and follow up ; Signficant < 0.05, Significant <0.01

	ΔBDI	ΔDHI	ΔHearing loss	ΔFacial paralysis	ΔVAS Dizziness	ΔHeadache
GBI total	0.184	0.235	0.218	0.455	0.071	0.042
GBI general	0.117	0.212	0.191	0.489	0.096	0.093
GBI social support	-0.092	-0.152	0.138	-0.021	-0.056	-0.112
GBI bodily health	0.189	0.333	0.269	0.054	-0.040	-0.176

	PF	RP	BP	GH	VT	SF	RE	PS
GBI total	0.092	0.142	-0.121	0.415	0.060	0.197	0.093	0.170
GBI general	0.085	0.126	-0.113	0.399	0.086	0.172	0.064	0.161
GBI social support	-0.324	-0.047	-0.264	-0.047	-0.080	0.015	0.059	-0.094
GBI bodily health	0.330	0.115	0.193	0.419	0.016	0.040	-0.064	0.110

Supplemental Online Table 6: Spearman correlation between change in functional measures and change in SF-36 subscores (uncorrected) between baseline (before surgery) and follow up; Significant < 0.05, Significant < 0.01.

	ΔPF	ΔRP	ΔBP	ΔGH	ΔVT	ΔSF	ΔRE	ΔPS
ΔHearing loss	0.000	-0.120	-0089	0.214	-0240	-0.292	-0.163	-0.253
ΔFacial paralysis	-0.039	-0.007	-0.092	0.300	0.093	0.237	0.278	0.242
ΔVAS Dizziness	0.303	0.308	0.207	-0.008	0.238	0.251	0.152	0.109
